# Bioremediation of heavy metals and petroleum hydrocarbons in diesel contaminated soil with the earthworm: *Eudrilus eugeniae*

**DOI:** 10.1186/s40064-015-1328-5

**Published:** 2015-09-22

**Authors:** Ogheneruemu Abraham Ekperusi, Iruobe Felix Aigbodion

**Affiliations:** Environmental Quality Management Programme, Department of Animal and Environmental Biology, Faculty of Life Sciences, University of Benin, Benin city, Nigeria

**Keywords:** Vermiremediation, Diesel, Earthworms, *Eudrilus eugeniae*, Nigeria, Niger delta, Heavy metals, TPH, BTEX

## Abstract

A laboratory study on the bioremediation of diesel contaminated soil with the earthworm *Eudrilus eugeniae* (Kingberg) was conducted. 5 ml of diesel was contaminated into soils in replicates and inoculated with *E. eugeniae* for 90 days. Physicochemical parameters, heavy metals and total petroleum hydrocarbons were analyzed using AAS. BTEX in contaminated soil and tissues of earthworms were determined with GC-FID. The activities of earthworms resulted in a decrease in pH (3.0 %), electrical conductivity (60.66 %), total nitrogen (47.37 %), chloride (60.66 %), total organic carbon (49.22 %), sulphate (60.59 %), nitrate (60.65 %), phosphate (60.80 %), sodium (60.65 %), potassium (60.67 %), calcium (60.67 %), magnesium (60.68 %), zinc (60.59 %), manganese (60.72 %), copper (60.68 %), nickel (60.58 %), cadmium (60.44 %), vanadium (61.19 %), chromium (53.60 %), lead (60.38 %), mercury (61.11 %), arsenic (80.85 %), TPH (84.99 %). Among the BTEX constituents, only benzene (8.35 %) was detected in soil at the end of the study. Earthworm tissue analysis showed varying levels of TPH (57.35 %), benzene (38.91 %), toluene (27.76 %), ethylbenzene (42.16 %) and xylene (09.62 %) in *E. eugeniae* at the end of the study. The study has shown that *E. eugeniae* could be applied as a possible bioremediator in diesel polluted soil.

## Background

Activities in the over half a century operations in the oil and gas industry in Nigeria has resulted in unprecedented release of hydrocarbons and associated pollutants including heavy metals into the Niger Delta environment from refine and unrefined petroleum products (Obot et al. 2006; UNEP 2011). The severe pollution scenario has resulted in the degradation of the environment especially soil, resulting in a substantial decline in both below and above ground biodiversity, affect public health and disrupt life support system for local people.

Diesel, a product of the fractional distillation of crude oil has the potential and associated health effects with crude oil. Although, there are a wide range of constituents in diesel as well as crude oil, research in recent years has focused on the total petroleum hydrocarbons and associated compounds like BTEX (Salanitro et al. 1997) due to their carcinogenic and mutagenic properties (EPA 2012) and the possibility of several other diseases such as white blood cell count (Kirkeleit et al. 2006; Lewander and Aleguas 2007) Hodgkins lymphoma, aplastic anemia, acute leukemia, bone marrow abnormalities (Kasper et al. 2004) and myelodysplastic syndrome (Smith 2010; IARC 2012). These considerable health effects have made government, industry and scientists to sustain the zeal to find novel approach towards restoring polluted environment.

The quest for environmentally sustainable approach towards contaminated environment has made the ever evolving field of bioremediation a standard practice for the remediation and restoration of degraded environment. Over the years, considerable research has been published in the area of bioremediation with diverse modifications for the treatment of a wide range of contaminants in both laboratory and field studies (Dash 1978; Hutchins et al. 1992; Hoff 1993; Adriano et al. 1999; Singer et al. 2001; Barker and Bryson 2002; Cheng and Wong 2002; Singleton et al. 2003; Tharakan et al. 2004; Tomoko et al. 2005; Zorn et al. 2005; Ceccanti et al. 2006; Chris 2007; Hongjian 2009; Iordache and Borza 2012).

Despite the success recorded so far, attempt to elucidate appropriate and effective bioremediation protocol for petrochemicals has remain inconclusive and contradictory (Ma and Imerzeel 1995; Schaefer and Filser 2007; Singer et al. 2001; Ameh et al. 2013). The composition, geological formation and types of hydrocarbons found in different regions of the world makes it almost impossible to apply a uniform approach in the bioremediation of crude oil and associated constituents. This realization has created the need for researchers to find appropriate and suitable ecological based approach in each region for the bioremediation of petroleum hydrocarbons.

Literatures on the bioremediation of diesel contaminated soil with earthworms are quite few, hence this study aims to explore the potentials of *Eudrilus eugeniae* on the bioremediation of heavy metals and hydrocarbons in diesel contaminated soil.

## Results

### Physicochemical parameters

**Fig. 1:**
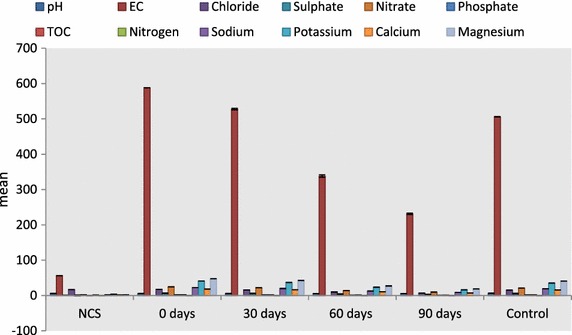
Physicochemical parameters of diesel contaminated soil with *E. eugenia*

The physicochemical parameters of the soil increased significantly after contamination with diesel at day 0 (Fig. 1) but decreased significantly after inoculation with *Eudriluseugeniae* within 30, 60 and 90 days. pH decreased by 1.40, 3.0, 2.62 % (F = 177.24 P < 0.05), followed by electrical conductivity 10.26, 42.46, 60.66 % (F = 11,969.70, P < 0.05), total nitrogen 26.32, 36.84, 47.37 % (F = 49.00, P < 0.05), chloride 10.32, 42.46, 60.66 % (F = 2233.94, P < 0.05), total organic carbon 26.32, 37.70, 49.22 % (F = 294.55, P < 0.05), sulphate 10.36, 42.50, 60.59 % (F = 10,794.72, P < 0.05), nitrate 10.20, 42.51, 60.65 % (F = 10,949.60, P < 0.05), phosphate 10.23, 42.05, 60.80 % (F = 5083.53, P < 0.05), sodium 10.79, 42.35, 60.65 % (F = 8549.64, P < 0.05), potassium 10.30, 42.23, 60.67 % (F = 12,267.98, P < 0.05), calcium 10.37, 42.29, 60.67 % (F = 11,989.62, P < 0.05) and magnesium 10.23, 42.98, 60.68 % (F = 7944.00, P < 0.05).

### Heavy metals

**Fig. 2:**
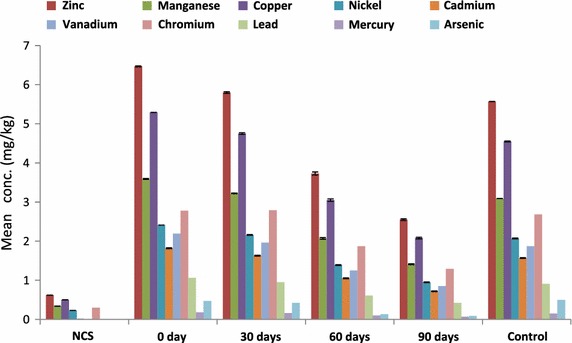
Heavy metals bioremediation in diesel contaminated soil with *E. eugeniae*

**Fig. 3:**
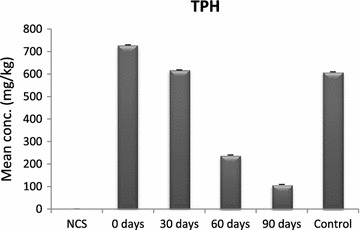
Bioremediation of TPH in diesel contaminated soil with *E. eugeniae*

There was a corresponding significant decreased in the heavy metals concentration with *E. eugeniae* (Fig. 2) after 30, 60 and 90 days of the study. Zinc decreased by 10.36, 42.35, 60.59 % (F = 10,978.29, P < 0.05), manganese 10.31, 42.34, 60.72 % (F = 12,986.98, P < 0.05), copper 10.21, 42.34, 60.68 % (F = 12,301.63, P < 0.05), nickel 10.37, 42.32, 60.58 % (F = 11,201.04, P < 0.05), cadmium 10.44, 42.31, 60.44 % (F = 9149.73, P < 0.05), vanadium 10.50, 42.92, 61.19 % (F = 10,524.67, P < 0.05), lead 10.38, 42.45, 60.38 % (F = 2549.33, P < 0.05), mercury 11.11, 44.44, 61.11 % (F = 812.91, P < 0.05), arsenic 10.64, 72.34, 80.85 % (F = 82.50, P < 0.05) while chromium initially increased by 0.36 % after 30 days and then decreased by 32.73 and 53.60 % after 60 and 90 days (F = 53.25, P < 0.05) of the study (Fig. 3).

### Bioaccumulation factor (BAF) for heavy metals

For this study, bioaccumulation factor was evaluated as the concentration of the heavy metals in the earthworms in relation to the concentration in soil after 90 days of the experiment.

$${\text{BAF}}_{\text{heavy metals}} = \frac{HM_E}{HM_S}$$

where

BAF_heavy metals_ = bioaccumulation factor for heavy metals

*HM*_*E*_ = heavy metal concentration in the earthworms (mg/kg)

*HM*_*S*_ = heavy metal concentration in the soil (mg/kg)

The BAF for heavy metals in the contaminated soil showed that zinc, copper, nickel, had a BAFs of 1.54, while manganese, cadmium, vanadium, chromium, lead, mercury and arsenic has a BAFs of 1.55, 1.53, 1.58, 1.20, 1.52, 1.57 and 4.22 respectively.

### Total petroleum hydrocarbon

After inoculation of diesel contaminated soil with *E. eugeniae* the total petroleum hydrocarbons decreased by 15.20 % after 30 days, 67.08 % after 60 days and 84.99 % after 90 days, for the control, it decreased by 16.41 %. Analysis of variance showed a significant difference (F = 28,614.53, P < 0.05).

### Benzene, toluene, ethylbenzene and xylene (BTEX)

**Fig. 4:**
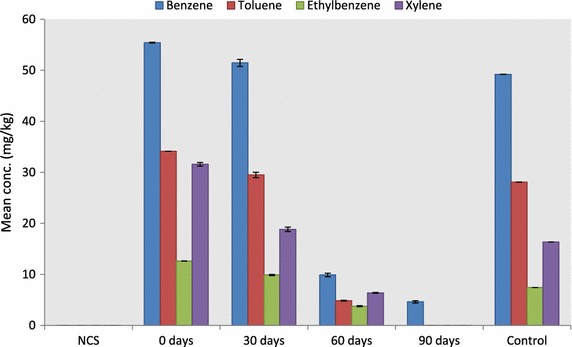
Bioremediation of BTEX in diesel contaminated soil with *E. eugeniae*

The rate of bioremediation for the BTEX components is shown in Fig. 4. Benzene decreased by 7.18, 82.16, and 91.65 % (F = 6570.14, P < 0.05), toluene by 13.65, 85.77 and 100.00 % (F = 5468.281, P < 0.05), ethylbenzene by 21.63, 70.13 and 100.00 % (F = 6115.70, P < 0.05) and xylene by 40.10, 79.66 and 100.00 % (F = 6.55, P < 0.05) after 30, 60 and 90 days of the study.

### Earthworm bioaccumulation/biodegradation in contaminated soil

**Fig. 5:**
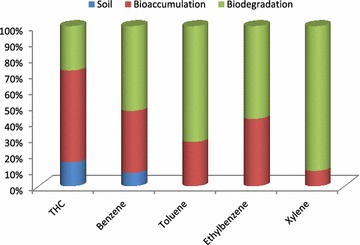
Earthworm bioaccumulation/biodegradation in diesel contaminated soil with *E. eugeniae*

At the termination of the experiment, earthworms were taken for analyses using GC-FID to estimate the rate of bioaccumulation and biodegradation (Fig. 5) of TPH and BTEX by *E. eugeniae*.

**Fig. 6:**
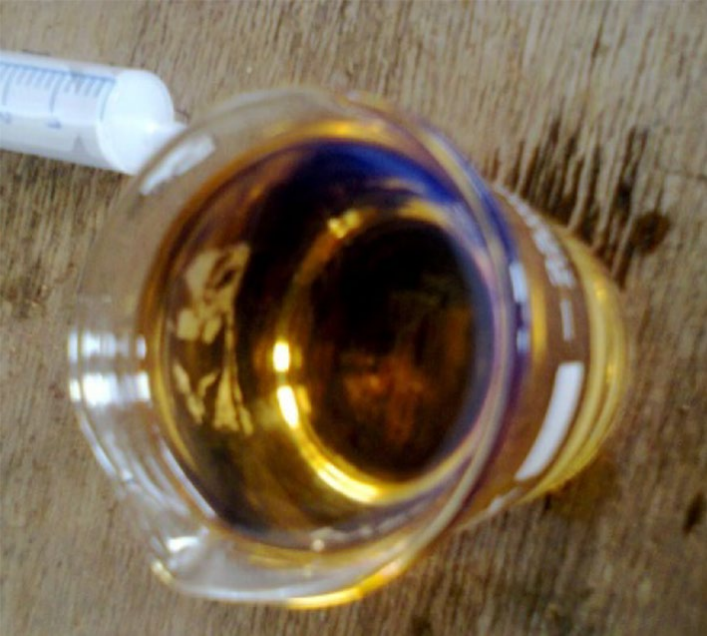
Diesel oil used for the experiment

**Fig. 7:**
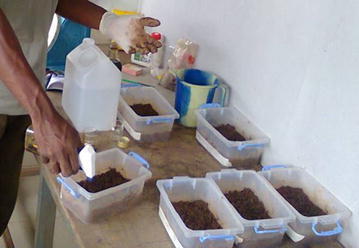
Researcher, preparing the experimental setup

**Fig. 8:**
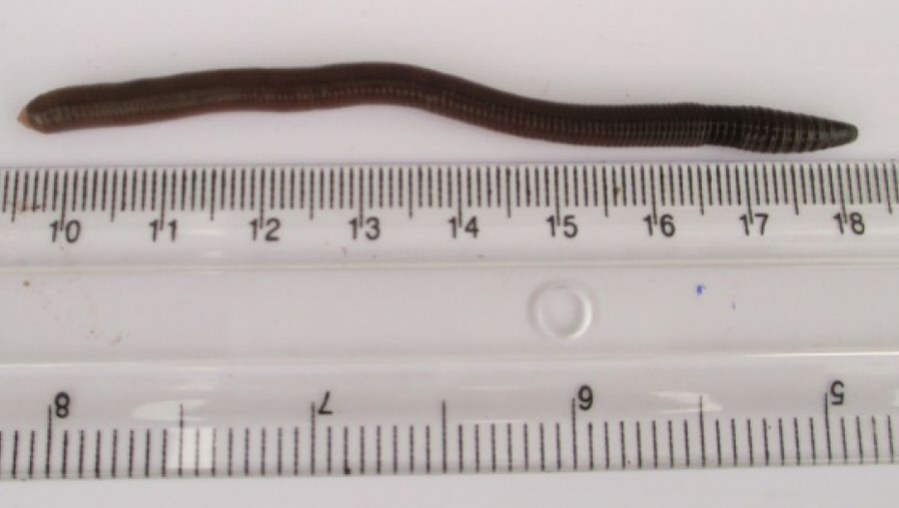
*Eudrilus eugeniae*

Analyses of the tissues of *E. eugeniae* at the end of the experiment showed that the earthworm bioaccumulated 57.35 % TPH, 38.91 % benzene, 27.76 % toluene, 42.16 % ethylbenzene and 09.62 % xylene (Figs. 6, 7, 8).

Method described by Ekperusi and Aigbodion (2015) was adopted for estimating the biodegradation of TPH and BTEX as stated below

$${\text{Biod}}_{\text{C}} {\text{ = I}}_{\text{C}} - {\text{ F}}_{\text{C}} - {\text{ T}}_{\text{C}}$$

where:

Biod_C_ = Concentration of the pollutant biodegraded at the end of the experiment

I_C_ = Initial concentration of the pollutant at the beginning of the experiment

F_C_ = Final concentration of the pollutant at the end of the experiment in the media

T_C_ = Concentration on the tissues of the earthworms at the end of the experiment.

The results revealed that the earthworms *E. eugeniae* biodegraded 27.64 % TPH, 52.73 % benzene, 72.24 % toluene, 57.85 % ethylbenzene and 90.38 % xylene.

## Discussion

Earthworms are fast becoming a choice organism for bioremediation of a wide range of contaminants from polluted soil environment. In this study, *E. eugeniae* survived the concentration of diesel applied, favourably altered and enhanced the physicochemical conditions of the polluted soil, significantly reduced the concentration of heavy metals, total petroleum hydrocarbons and benzene while toluene, ethylbenzene and xylene were eliminated from the polluted soil.

Careful observation revealed that the earthworms survived the diesel concentration and were able to adapt and carry out their activities inside the polluted soil. This is consistent with the report of Sun et al. (2011) that observed no mortality of earthworms on the bioremediation of pyrene with *E. foetida*, but contrasted that of Contreras-Ramos et al. (2006) with 80 % survival of *E. foetida* in soil amended with anthracene and benzo(a)pyrene. The variation in mortality observed maybe due to the different concentration and composition of petrochemicals applied in the research. At the end of our study, earthworms re-introduced back into non-polluted soil continue their normal biological activities. It should be noted that bioremediation was more effective after 60 and 90 days of the study. This could be attributed to the fact that *E. eugeniae* needed sufficient time to adjust to the polluted environment before bioremediation sets in.

Physicochemical characteristics of polluted soil decreased significantly with the inoculation of earthworms. The decrease probably coincided with heavy metals elimination from polluted soil and hydrocarbon degradation. Ceccanti et al. (2006) reported a decrease in carbon, phosphorus and C:N in all treatments, suggesting a progressive degradation of pollutants.

pH is very essential in regulating the physical and chemical conditions of soil and affects the mineral constituents of soil available to the functioning of soil organisms. The pH of soil decreased after contamination and decreased further after inoculation with *E. eugeniae*, within 30 and 60 days, but slightly increased after 90 days. The factor responsible for this trend is not clear, but this variation in pH was not unusual since it is still within the acceptable pH range (Owa et al. 2003) for earthworms to carry out their biological function in polluted soil. It has been reported that the pH of soil materials decreased during gut passage in *Lumbricus terrestris* (Heine and Larink 1993).

Electrical conductivity of soil is a measure of the amount of salts in soil. It is an important indicator of soil health (NRCS 2013). EC of soil highly increased after contamination of soil and gradually decreased with the inoculation of earthworms. The decreased at the end of the study was still far higher than the background levels prior to contamination of soil. It is possible that the earthworms only have the capacity to utilize the desired levels of salts needed for the bioremediation process. An extended duration of the study may have resulted in further decrease of EC. Manyuchi et al. (2013) reported that soil EC decreased on increasing the vermicompost quantity while Ceccanti et al. (2006) reported a decrease in the EC of the soil in the first month, an increase in the second month before it decreases again in the third month.

Total organic carbon is the amount of carbon bound in an organic compound or geological formation (EIA 2013) while total nitrogen measures the total amount of nitrogen present in soil, much of it which is held in organic matter. Carbon and nitrogen decreased below the background levels at the end of the study. The accumulation and breakdown of pollutants by the earthworms may have affected the levels of carbon and nitrogen in the polluted soil since the earthworms may require some degree of these nutrients to maintain its biological function in the bioremediation process. Edwards and Arancon (2006) suggested that the breakdown of compost materials produced fully stabilized organic amendments with low C:N ratios.

Chloride in soil increased after contamination and decreased significantly below the background levels at the end of the study. There is the likelihood that the passage of contaminated soil across the gut of earthworms may have influenced the alteration of chloride leading to the decreased. Also, the activity of the earthworm’s calciferous glands (Salmon 2001) may have altered the levels of chloride in favour of the bioremediation process.

Sulphate, phosphate, nitrate, magnesium, sodium, calcium and potassium although decreased significantly but deviated from the observed trend with the other physicochemical characteristics of soil by being elevated above the background levels. It was suggested that the earthworms selectively enhance these parameters since they are vital nutrients needed for the enrichment of soil for soil flora and fauna. This help improved the quality of the soil for agricultural purposes. In a similar fashion, Sinha et al. (2009) reported that the earthworm increased vital minerals in soil enhancing the soil quality and nutrients.

Prior to contamination of soil, zinc, manganese, copper, nickel, and chromium were detected in very low concentrations in soil, while cadmium, vanadium, lead, mercury and arsenic were below detection limits. The heavy metals concentration in soil inoculated with earthworms showed a significant decreased after 30, 60 and 90 days of the experiment, although with variation. In the first 30 days of the study, manganese, chromium and arsenic remains unchanged, this is not unconnected with the possibility that *E. eugeniae* may have different response, adaptive mechanism and bioremediation approach to each heavy metals in polluted soil. At the end of the study, the heavy metals decreased in soil by 57 %. Sinha et al. (2009) reported most significant and rapid changes of over 80 % free of heavy metals in vermicomposted sludge with earthworms upon chemical analysis while Iordache and Borza (2012) reported a decreasing trend for lead, manganese, copper and zinc with lumbricoid earthworms.

On the effect of organic matter substrate on the toxicity of copper to *Aporrectodea longa* Ezemonye et al. (2006) reported that uptake increased as copper concentration increased in soil amended with organic substrates such as pig, poultry and cow manure.

Studies have documented that earthworms have the potentials to effectively bioremediate and detoxify heavy metals through several methods such as methylation, sequestration and growth dilution (Langdon et al. 2001). Metallothionein (MT) is a metal-binding protein thought to detoxify heavy metals in earthworms (Janssens et al. 2009). A preliminary study of *L. rubellus* has revealed the presence of MT2 protein around blood vessels in the chloragogenous tissue (Langdon et al. 2001). This may play a role in the detoxification and expulsion of arsenic and other heavy metals from contaminated earthworms (Langdon et al. 2001). The entire interaction of earthworms and heavy metals still remains uncertain. We can only begin to get a clear picture when more studies focus on earthworms exposed to a single heavy metal concentration under laboratory conditions.

The higher the bioaccumulation factors for a specific pollutant, the more the uptake. The uptake of zinc, copper and nickel followed the same trend—chromium had the lowest uptake while arsenic showed the highest uptake. The preference for the selective uptake of heavy metals by *E. eugeniae* is not known but the level of trace element requirement and rate of bioremediation may influence the levels of uptake of heavy metals by the earthworms. On the heavy metal accumulation by earthworms Dai et al. (2004) reported that the biota-to-soil accumulation factors (BSAFs) of the four heavy metals were ranked as cadmium > zinc > copper > lead.

Total petroleum hydrocarbon (TPH) was not detection in soil prior to contamination. TPH in the contaminated soil with earthworms decreased by 84.99 %, in contrast, that of the control without earthworms only decreased by 16.41 %. This reduction in TPH far exceeded that of Schaefer and Filser (2007) with 30–42 % in samples with *E. eugeniae* and 9–17 % without earthworms after 28 days. Ameh et al. (2012) reported 36.28 % TPH decrease with *E. eugeniae* after 42 days of treatment. The longer duration of 90 days in our study may have resulted in the higher reduction in TPH.

BTEX was not detected in soil prior to contamination with diesel. After contamination and inoculation with *E. eugeniae*, the levels of BTEX decreased significantly due to earthworm activities. At the end of the study, toluene, ethylbenzene and xylene were not detected in soil except benzene. On the bioremediation of crude oil with *H. africanus*, Ekperusi and Aigbodion (2015) reported the elimination of toluene, ethylbenzene and xylene from the contaminated soil leaving some fractions of benzene at the end of the study. Among the BTEX component of petroleum, benzene has been reported to be recalcitrant in bioremediation process.

In bioremediation research, it is essential that the transport and fate of pollutants should be critically assessed to ensure a comprehensive strategy for the removal of such pollutants from the environment (Ekperusi and Aigbodion, 2015). Although, earthworms biodegraded some levels of hydrocarbons from the polluted soil, at the termination of the study, *E. eugeniae* were shown to bioaccumulated 9.62 % xylene, 27.76 % toluene, 38.91 % benzene, 42.16 % ethylbenzene and 57.35 % TPH. Tharakan et al. (2004) and Contreras-Ramos et al. (2006) reported elevated amount of PCBs and PAHs in earthworm biomass at the end of their studies. It is highly probable that the earthworms needed a considerable time beyond the design of our study to degrade the entire contaminants in its tissues. It is also possible that some constituents of hydrocarbons are recalcitrant to transformation (Tharakan et al. 2004) by earthworms.

It is our favoured opinion that there is the likelihood that the earthworm first bioaccumulates and immobilized pollutants to some extent in its tissues before biodegradation set in. The breakdown of pollutants in the tissues of earthworms may be triggered when the levels of the pollutants threatens the earthworm survival especially in confines or restricted environment. To what extent the earthworm can bioaccumulates pollutants and the triggering mechanisms for the breakdown of pollutant is a matter for further studies.

## Conclusion

Finding a sound ecological based approach towards the removal of petroleum hydrocarbons from the environment is one of the crucial issues in modern society. This study investigated the potentials of *E. eugeniae* on the bioremediation of heavy metals, total petroleum hydrocarbons and BTEX in diesel polluted soil under laboratory conditions.

Careful observations and analyses of the unpolluted soil, polluted soil and the tissues of the earthworms within the study period provided insights on how the earthworm *E. eugeniae* can bioremediate heavy metals and hydrocarbons from polluted soil, but the complex nature of the process remains a mystery. A thorough understanding of the interactions and fate of pollutants is highly needed to get a clearer picture of the full nature of earthworm bioremediation pathway in contaminated environment. From the outcome of this study, *E. eugeniae* could be applied for the bioremediation of moderately polluted soil with petrochemicals.

## Methods

### Test substrate

The soil used for the experiment was collected besides the botanical garden of the Faculty of Life Sciences, University of Benin, Benin City. Top soil not exceeding a depth of five inches after clearing the vegetation cover was dug with a shovel and collected into a bucket. The collected soil was sun-dried by spreading it on a flat clean board surface for 48 h. Thereafter, the sun-dried soil was sieve by using a 5 mm plastic mesh filter according to the ISO (1993) to remove debris and large stones.

### Test organisms

The earthworms used for the experiment, were collected around the main campus of the University of Benin. Earthworms were dug with shovel from soil under shaded areas at the Faculty of Life Sciences. After each collection, earthworms are kept in a holding container. All earthworms were held in the holding facility for 7 days prior to the experiment for acclimatization purposes. Earthworms for the study were identified using methods described by Owa (1992).

### Contaminants

The diesel for the research was purchased from a major petroleum marketer, in Benin City. Two litres of diesel was purchased in a container for the purpose of the research and transported to the laboratory.

### Experimental design

Four translucent rectangular plastic containers having strong cover lid with clips on two sides of the edges measuring 20 × 9 × 12 cm were purchased from the market. The inside of the cover lid were cut off leaving the frame of the lid. The containers were weighed with a digital sensitive weighing balance (Scoute SE, 410X0.019, Ohaus Computer, Parsippany, NJ, USA), while the soil was then weighed into 1 kilogram each using Camry Emperors (ISO 90001) scale manufactured by Dial Spring Scale, Guangdong, China, into each of the experimental containers.

### Contamination of soil

With the aid of a 10 ml glass beaker, 5 ml of diesel was thoroughly mixed, into each of the four containers with 1 kilogram of soil and were moistened with distilled water to the water holding capacity of the soil. The treatments were left to stay for 7 days in the laboratory exposed to the elements.

### Addition of organic additives

After 7 days, cow dung were collected fresh within the campus and about 50 g each of the cow dung weighed with a digital sensitive weighing balance, were thoroughly mixed as additives into the containers with diesel contaminated soil.

### Inoculation of test organism

Immediately after the addition of additives earthworms were sorted out from the holding containers, washed with clean water and ten earthworms of the species *Eudrilus eugeniae* measured and weighed were inoculated into each container with diesel contaminated soil and the replicates except the control. A netting material was placed on top of each of the containers and the cover lid frame was used to hold the containers firmly. This is done to avoid escape of the earthworms and to allow free flow of oxygen into the treatment in the course of the experiment. The setup was placed inside the laboratory and checked morning and evening on a daily basis for 90 days.

### Laboratory analyses

Prior to the contamination of the soil and after contamination, samples of the non-contaminated soil (NCS) were collected, placed in an aluminium foil, labeled with codes and transported to the laboratory to determine the physicochemical parameters, heavy metals, total petroleum hydrocarbon (TPH) and the benzene, toluene, ethylbenzene and xylene (BTEX) constituents (Table 1).

**Table 1:** Soil texture for the experiment

Parameters (%)	Non-contaminated soil
Sand	96.00 ± 0.00
Silt	3.00 ± 0.00
Clay	1.00 ± 0.00

For every 30 days, samples of the contaminated soil from each of the treatments were collected for laboratory analyses. For each of the contaminated soil, the physicochemical parameters such as pH, electrical conductivity, total organic carbon, chloride, nitrogen, sulphate, nitrate, phosphate, sodium, potassium, calcium, magnesium were determined using procedures by the AOAC (2005). Heavy metals and total petroleum hydrocarbons was determined using Atomic Absorption Spectrophotometer (AAS) as described by (Miroslav and Vladimir 1999) while benzene, toluene, ethylbenzene and xylene (BTEX) constituents were determine using Gas Chromatography with Flame Ionization Detector (GC-FID) from Agilent Technologies Inc., United States. At the termination of the experiment, earthworms were analyzed to determine the TPH and BTEX in the tissues using GC-FID.
